# Challenges and opportunities for policy decisions to address health equity in developing health systems: case study of the policy processes in the Indian state of Orissa

**DOI:** 10.1186/1475-9276-10-55

**Published:** 2011-11-18

**Authors:** Saji S Gopalan, Satyanarayan Mohanty, Ashis Das

**Affiliations:** 1 Abad Aquarius, Kochi, Kerala, Pin: 683105, India; 2 DCOR Consulting Private Ltd, 131(P), Satyanagar, Bhubaneswar, Orissa, Pin: 751007, India

**Keywords:** health equity, policy processes, health sector reforms, developing health systems, India

## Abstract

**Introduction:**

Achieving health equity is a pertinent need of the developing health systems. Though policy process is crucial for planning and attaining health equity, the existing evidences on policy processes are scanty in this regard. This article explores the magnitude, determinants, challenges and prospects of 'health equity approach' in various health policy processes in the Indian State of Orissa - a setting comparable with many other developing health systems.

**Methods:**

A case-study involving 'Walt-Gilson Policy Triangle' employed key-informant interviews and documentary reviews. Key informants (n = 34) were selected from the departments of Health and Family Welfare, Rural Development, and Women and Child Welfare, and civil societies. The documentary reviews involved various published and unpublished reports, policy pronouncements and articles on health equity in Orissa and similar settings.

**Results:**

The 'health policy agenda' of Orissa was centered on 'health equity' envisaging affordable and equitable healthcare to all, integrated with public health interventions. However, the subsequent stages of policy process such as 'development, implementation and evaluation' experienced leakage in the equity approach. The impediment for a comprehensive approach towards health equity was the nexus among the national and state health priorities; role, agenda and capacity of actors involved; and existing constraints of the healthcare delivery system.

**Conclusion:**

The health equity approach of policy processes was incomprehensive, often inadequately coordinated, and largely ignored the right blend of socio-medical determinants. A multi-sectoral, unified and integrated approach is required with technical, financial and managerial resources from different actors for a comprehensive 'health equity approach'. If carefully geared, the ongoing health sector reforms centered on sector-wide approaches, decentralization, communitization and involvement of non-state actors can substantially control existing inequalities through an optimally packaged equitable policy. The stakeholders involved in the policy processes need to be given orientation on the concept of health equity and its linkage with socio-economic development.

## Introduction

Health equity is a situation where physical, financial, and managerial resources are adequately available to enable every individual a healthy living. In order to achieve this, the resources need to be distributed optimally to cater to various determinants of health (nutrition, housing, water, sanitation, livelihoods etc.) apart from healthcare [1]. Such a resource distribution can offset the existing inequalities in the health status of populations, their future emergence and recognize being healthy as a human right. Globally, achieving health equity is still a far-fledged target for many health systems, despite significant achievements in overall health status of populations and health systems indicators [2]. Meeting the Millennium Development Goals and other health targets urge for addressing health equity and thereby inclusive socio-economic development [3]. Attaining an equitable health system depends upon baseline health systems characteristics and comprehensive systemic attempts to address inequality [4,5]. Health inequity is more a challenge in developing countries like India due to; presence of huge inequalities; recognized bi-directional relationship between inequality and inequity; and less systemic preparedness to address inequity due to resource constraints including lack of awareness [6].

An equitable approach in health policy is a necessity in developing countries due to the public good characteristics of healthcare and risk of information asymmetry [7,8]. There are certain technical (ideologies, knowledge etc.) and non-technical (funds, infrastructure etc.) determinants of an equitable policy approach propelled by global, national and regional factors [9]. 'Path dependency', where the legacy of policy structure makes divergence difficult, is a case for technical impediment; while, the existing inadequate infrastructure hindering all-encompassing approach represents a non-technical barrier [10]. This case study presents the synthesis of a policy process analysis of health equity, its challenges and opportunities in the Indian State of Orissa. The study outcomes are expected to facilitate improved policy decisions towards health equity in Orissa and similar settings.

### Context for health equity case study in Orissa

The developing Indian health system exhibits a co-existence of improving health indicators and widening socio-economic and health disparities [11]. Orissa is a less developed Indian State possessing relatively adverse socio-economic and health systems profile as shown in Table 1. The Indian health policy approach has clearly demonstrated a considerable focus on an 'equitable healthcare system' starting from the Bhore Committee Report (1948) to the recent National Rural Health Mission (NRHM) [12,13]. Yet, India lacks substantial evidences on tracking the policy processes on health equity as many other developing countries [1]. Policy processes stemmed from globalization and structural adjustment programs need to be evaluated as they are known to introduce inequity [14]. For instance, public-private partnerships and sector-wide approaches are considered as double-edged swords and could be inequitable, if not ascertained adequately [15].

**Table 1:** A comparative assessment of the crucial socio-economic development indictors of Orissa and India

**Sl. No**.	Indicators	Orissa	India
1	Area (Sq. Km.)	155,707	3,287,263
2	Total Population	36,804,660	1,028,610,328
	2.1 Rural Population (%)	85.01	72.18
	2.2 Scheduled Tribe Population (%)	22.13	8.20
3	Disabled Population (%)	2.8	2.1
4	Population Density (per sq. km.)	236	313
5	Sex Ratio (females per 1,000 males)	972	933
6	Total Literacy (%)	63.1	64.8
	6.1 Male Literacy (%)	75.3	75.3
	6.2 Female Literacy (%)	50.5	53.7
7	Life Expectancy of Males at Birth (in yrs)	60.05	63.87
8	Life Expectancy of Females at Birth (in yrs)	59.71	66.91
9	Infant Mortality Rate	64.7	57.0
10	Maternal Mortality Ratio	358	301
11	Malnourished Children Below Five Years (Underweight)	41	43
12	Prevalence (%) of any anemia in children < 11.0 g/dl (6-59 months)	65.0	69.5
13	Prevalence (%) of any anemia in adult women < 12.0 g/dl (15-49 yrs)	61.2	55.3
14	Families living Below Poverty Line (%)	66.37	40.00
15	Poverty Ratio (Rural)	46.9	28.1
16	Poverty Ratio (Scheduled Tribe)	75.8	44.7

Sources: Government of India and Government of Orissa

This review on Orissa could be a useful case study to analyze how a resource constraint health system can address health equity through health sector reforms. Orissa's health sector witnessed the formulation of an 'Integrated Health Plan' (2002) and 'Orissa Health Sector Plan' (OHSP). The latter encompasses a package of specified targets, concentrated indicators and sector wide reforms to achieve the goals of the former [16]. OHSP is underway (2007-2012) with the support from the British Department for International Development (DFID). This case study aimed at bringing in evidences to augment the Government of Orissa's health sector reform process vis-à-vis health equity through OHSP. The study syntheses are relevant and have emerged out of analyzing the existing dynamics of a federal health system structure, health sector reforms, multi-sectoral nexus of equity and a developing health system.

### Methodology

**Figure 1:**
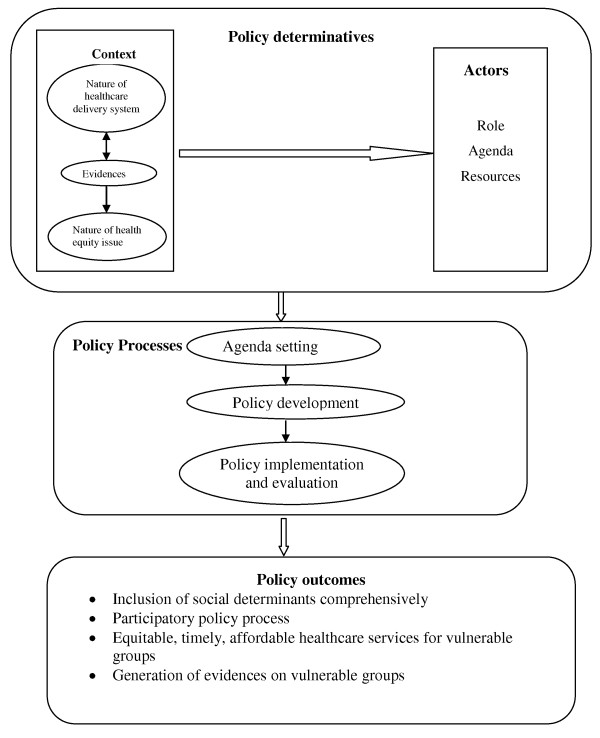
**Health equity policy process analysis framework**.

The specific objectives of the review were to understand the extent of 'equity approach' in the policy processes, its network and contextual determinants, opportunities and challenges. By 'health equity approach' we meant the intent and the ability of the policy decision to cater to the comprehensive health needs of vulnerable groups to ensure equitable health status of populations. We used the framework of health policy process analysis, derived from 'Walt and Gilson Policy triangle' with contextual modifications [17]. The framework as shown in Figure 1 consisted of analyzing the equity approach in policy processes i.e. agenda setting, policy development, implementation and evaluation. Since the specific focus was on Integrated Health Policy, the policy processes were analyzed after its formulation i.e. 2003-2009. The following were the contents of policy processes analysis framework.

1. Equity policy processes: What were the policies and how were they made at the following stages?

a. Agenda setting - equity vision, goals, and structure in health policies. We explored how the Integrated Health Plan of Orissa addressed 'equitable health status of populations' in its vision, goal and structure, and the constraints in this regard.

b. Policy development - equity strategies and action plans. We assessed how the Orissa Health Sector Plan strategized addressing 'equitable health status of populations' in the organization and management of healthcare delivery and public health interventions.

c. Implementation and evaluation - how the strategies and action plans were implemented and evaluated?

2. Policy determinatives: What were the determinants of policy processes?

a. Actors - who decided the agenda and strategies?

b. Context - micro and macro nature of the policy issue, i.e. health equity in the existing socio-economic, geo-political and health system dynamics

3. Policy outcomes: We concentrated on the following four major equity policy outcomes based on the historical arguments for equity. In order to achieve 'equitable health status of populations' or health equity, we analyzed the attempts to achieve these outcomes in the above policy processes

• inclusion of social determinants comprehensively

• inclusion of participatory policy processes

• generation and use of evidences on vulnerable groups and health inequalities

• equitable, timely, acceptable and affordable healthcare services for vulnerable groups

### Data collection

This case-study gathered information through a qualitative assessment consisting of key informant interviews (KII) and document reviews. Both KIIs and document reviews explored; the current equity approaches, measures and outcomes of various departments or programs; how the equity policies are designed; and the factors influencing the equity policy processes.

#### Key informant interviews (KII)

We interviewed major stakeholders from the departments of Health and Family Welfare, Rural Development, and Women and Child Welfare, and civil societies during the second half of 2009. The key informants (n = 34) at sub-national and district levels were identified based on their positions, roles and responsibilities on activities regarding policy processes and health equity. The key informants were instrumental either in the design of policies or their implementation and/or evaluation. The interviewees were administrators (n = 13), program managers (n = 12), and physicians (n = 5) of the government departments and civil society organizations (n = 4). The mean age and years of work experience were 42 and 14 respectively. The KIIs were conducted in English and Oriya (local language) by the primary author and each interview took around 20-40 minutes. The interviews were recorded with an electronic voice recorder. A pre-tested and semi-structured interview guide directed the interviews. The guide included actors' understanding about health equity and its need, current approach on health equity and their perceptions, their interests on policy decisions on health equity, challenges and opportunities.

#### Document reviews

The review involved various published and unpublished reports, policy pronouncements and articles on health equity in Orissa. The principal policy review centered on overall developmental plans, Orissa Health Sector Plan (OHSP), Vision 2010 (a document envisioning the health sector reforms in Orissa), NRHM mission document, NRHM Program Implementation Plans (national, sub-national and districts). While, other reviewed policy documents consisted of budget books, behavior change communication strategies, human resources and capacity development strategies, monitoring and evaluation framework. The secondary sources of information included annual reports of the Departments of Health and Family Welfare, Women and Child Welfare, Rural Development, and reports of national and district level health surveys.

### Data analysis

Transcription and translations (from Oriya to English) were performed, followed by entry into a word processing software (Microsoft Word). The transcripts were coded through NVivo 8 software and emergent themes were identified from the codes. Informed consent was obtained from all participants after explaining them the objectives of the study. Participation was voluntary and the key informants had the option to deny answering any question or withdraw at any point of time. Names of the respondents were removed during transcription and confidentiality was maintained throughout the study.

We used the 'grounded theory approach'^i ^to validate the opinions of major stakeholders on their perceptions and experience on health equity through the literature review. The document review involved an 'archival analysis'^ii ^of the socio-economic and political scenario of the state.

## Results and Discussions

If liberty and equality, as is thought by some are chiefly to be found in democracy, they will be best attained when all persons alike share in the government to the utmost... ... Aristotle, the Greek Philosopher

This section synthesizes the findings of the policy processes review during 2003-2009. In short, policy documents were embodied in plans and programs, legislations and projects.

### 1. Equity approach in policy processes

#### a. Agenda setting

The State of Orissa designed an equity centered 'integrated health policy' in 2002, which is one of the unique attempts among developing states in India [18]. It envisages for "*facilitating improvement in the health status of the people of Orissa with their participation, and to make available health care in a socially equitable, accessible and affordable manner within a reasonable timeframe, creating partnerships between the public, voluntary and private health sector and across other developmental sectors."*

While analyzing the intent on the four policy outcomes in the 'agenda setting', we found that social inclusion was the corner stone as seen in the above manifesto. There was a special mention of vulnerable groups based on geographical accessibility (hard-to-reach or inaccessible areas), social status (scheduled tribes and caste), gender (women) and economic status (below poverty line population). Almost all health systems prioritize on addressing the issues of such populations, which is reinforced through the 'Alma Ata Declaration' and the 'United Nations Millennium Declaration' [2,3]. In Orissa, notable features in the policy agenda in this regard were focus on primary healthcare of populations and the intent to blend 'health care delivery' with 'public health goods' (e.g. nutrition, sanitation etc.). This need for blending social development and health improvements is a rare realization in most of the policy goals in developing countries [18].

The policy agenda also specified about decentralized, participatory and reflexive approach in planning for equity, generation and use of evidences on inequalities, and essential healthcare for the marginalized. These are promising steps to address the comprehensive issues of the marginalized [8].

We observed certain constraints in the policy outlook vis-à-vis health equity. First, in the policy outlook, there was a missing link to 'acceptable care'. In a democratic health system, since health is a right, people should have acceptance for the services they receive [8,19]. Secondly, though the focus is more on socially backward groups, it did not address their comprehensive determinants of health. For instance, addressing their poverty related issues and cultural beliefs hindering healthy life were not looked upon. Similarly, focusing largely on socially backward groups (e.g. tribal communities) lead to the neglect of other determinants of general population to some extent as explained below. A right blend of health determinants was missing since addressing the socially backward groups alone is not sufficient, as populations have health risks owing to other social, medical and environmental susceptibility to illnesses [20]. For instance, Orissa has nearly 66% populations below the poverty line, which includes socially well-off groups as well [11]. Similarly, the state possesses considerable habitations near industrial plants facing environmental susceptibility to asthma, other respiratory illnesses and occupational health hazards [11]. Yet, this issue was substantially unnoticed in the policy agenda. In case of medical determinants, though disability is considered a matter of 'physical status,' still disabled were more approached from an 'economic vulnerability' point of view and was provided with only some financial assistance. But their medical determinants or risks of specific treatments and physical limitations of accessing institutionalized care were disregarded [11]. An absence of ramps in health facilities and a lack of strategies to provide them are some of the examples in this regard. Only by linking the social determinants right on the medical aspects, disease prevention and control can be managed effectively [8].

#### b. Policy development

Among various policy outcomes analyzed, OHSP's prime focus is ensuring healthcare delivery to unequal populations, which seems to be a part of equity-centric features of any health system [21-23]. Specific strategies existed for generating additional resources and provide innovative and differential service delivery (e.g. outreach health camps for hard-to-reach areas and subsidies or financial incentives to augment service utilization). Additional financial and techno-managerial resources were expected to generate from private and voluntary sectors like NGOs, philanthropies, care providers etc. OHSP also aims at collecting disaggregated health indicators for better planning for the marginalized.

However, we noted some limitations in this second stage of policy process. There was a slight dilution in the comprehensive approach towards marginalized groups from 'integrated health policy' (agenda setting) to OHSP (policy development). Bracketing all marginalized groups into the policy plan alone is not sufficient to address them, as their comprehensive needs are left out. The missing link to 'life-course approach' of women is a fine example here [24]. The scope of social determinants was confined to sanitation and nutrition, and neglected the crucial link of livelihoods and poverty on ill-health [8]. Though 66% of the population lived below the poverty line, the policies omitted strategies to enhance affordability and financial access to care largely [8]. A comprehensive financial protection measure was not a part of health policies, except some incentives to boost healthcare and exemption from user fee for some population groups. Further, though required, strategies on essential service packages and minimum standard for care were given less priority [25,26].

One of the reasons for these lacunae was the conservative approach of the policy formulation, underpinning the institutional agenda of the federal and state governments. For instance, the equitable healthcare delivery approach was centered on the 'vertical programs', which have a 'disease centric approach', narrowing down the scope of public health interventions [2]. Each vertical disease or health program has its own target population; some of them are at risk of diseases, inadequate service delivery or a combination of both. Therefore, such programs could not cater to all those who have adverse social determinants of health.

*"Though we have a health policy agenda, we confine to the overarching goals of the national health policies, programs and strategies." *[a state level stakeholder]

The 'path dependency', also made the innovative approaches in strategizing sub-optimal. Context-specific planning was hindered by adherence to the overarching national or state agenda, lack of confidence to approach unconventional ways and techno-managerial inadequacy, despite the involvement of bottom level stakeholders in health strategizing [27].

*"Going out-of-track of what has been happening or planned is risky. Changes are required, but it should not be in haste." *[a state level stakeholder]

*"We want to cater to populations residing in forest villages, but it needs unconventional approaches. We mostly try for innovation within the prescribed lines."[*a district level stakeholder]

The strategies were not specific on how to materialize the equity goals. For instance, the plan aimed at equitable allocation, but did not mention what should be the criterion for equitable allocation. Likewise, the convergence with allied sectors also remained clueless on materialization as strategies were lacking on how and for what the integration was intended for.

#### c. Policy implementation and evaluation

The policy implementation and evaluation process met with the same kind of leakage as that of policy formulation process. The two major leakages were on non-comprehensive coverage of marginalized groups and their needs, and ignorance of the provision of public goods largely. For instance, populations at risk of occupational and industrial health hazards, elderly, disabled, and economically backward populations belonging to higher social groups were omitted with no specific healthcare plan for them. Though the identification criteria for vulnerable groups should be apt and inclusive, yet developing health systems like Orissa find it difficult to map them owing to sparse evidences [28,29]. We noticed a trend of over- reliance on flagship programs to address health equity, which may not bring in a comprehensive and long term solution for the issues of the marginalized.

"*Health department cannot provide all public goods to enhance health equity, an objective oriented multi-sectoral integration alone can do it."*[a state level stakeholder]

*"NRHM has a number of programs to address the issues of the poor, otherwise, while dealing with diseases, it would be difficult." *[a state level stakeholder]

*"We try to address equity aspects within the framework of NRHM, but it has its own limitations as it is mainly intended for reproductive and child health issues." *[a state level stakeholder]

Resource generation to enhance service delivery could not tap optimally the potentials of the private sector, philanthropic sources and corporate social responsibility, as there existed a very few partnerships with them [11]. Some strategies were unrealistic for the stakeholders and they expected specific guidelines on implementation. A district level stakeholder opined on their cluelessness on how to generate and utilize resources in decentralized management of health centers. The absence of differential approaches for the physically challenged and funds for emergency care in the presence of a large number of poor illustrate this ignorance among the stakeholders.

The existing limited evidences on health determinants and outcomes of different populations further crippled an informed planning and strategizing on health inequalities. HMIS captured background information on age and gender; while socio-economic, occupational and educational status are mostly ignored. Apart from the national household surveys on health (though not annually), there were hardly any attempt on capturing health inequities or inequalities through baseline surveys or impact evaluation of programs and health policies.

*"The current 'health management information system (HMIS)' is not ripe enough to capture adequate disaggregated information on vulnerable groups and omits crucial information on socio-economic backgrounds due to non-feasibility*."[a district level stakeholder]

### 2. Policy determinatives

#### Actors and Context

The major actors involved in the policy planning were the national, state and district governments, donors and development agencies with limited participation from the civil society. The general planning framework of the health programs were initiated by the federal actors (policy makers, donors and civil society organizations at the national level) in due consultations with the state level stakeholders. Though decentralized planning was expected in principle, it confined to the overarching agenda of the federal and state governments. For instance, the state government followed the guidelines of the national government for the annual planning, implementation and monitoring of the NRHM programs, while the district level governments followed the state government. The presence of civil society organizations represented by a large group of people's movements in various social sectors (e.g. health, education, nutrition, human rights etc) was mostly at the national level planning. The approach to integrate healthcare delivery and public health interventions (nutrition, sanitation etc.) excluding other social determinants (e.g. poverty, housing, living conditions etc.) by the donors was one of the reasons for non-comprehensive approach towards health equity. The lack of integration between the departments on social and public policy also constrained a comprehensive approach on health equity. This subsequently led to insufficient and inappropriate strategies on healthcare delivery of the vulnerable and data for decision making.

*"I do not know to what extent the issues of the needy are the focus of health policies. Civil societies are involved for name sake in the health planning. Sometimes, we are invited to be part of the district and block level health planning. But, I feel civil societies should be given more participation at the state level planning."*[a civil society member]

*"We try to incorporate grass roots NGOs in the annual action plan. Otherwise, the civil society participation is at the National level."*[a state level stakeholder]

*"While designing the actions plans at the district level, we have been directed to follow the overall state agenda and approach. However, we get opportunities to have specific local plans within such overall agenda." *[a district level stakeholder]

*"We have a tradition of having a specific development issue with one department. The donor support does not intend to link the health of populations with the social development." *[a state level stakeholder]

*"It is true that currently, the government system has been re-designed for improving maternal and child health. I feel, people's other health issues, especially those of women should be prioritized." *[a stakeholder from a development agency]

The existing inhibition and lack of awareness among stakeholders on the link between health and social development restricted the inter-sectoral convergence on combating health inequities. For instance, most of the physicians interviewed considered health as more of a matter of medical science than a collective outcome of socio-medical determinants, though they are crucial for a healthy living [30,31]. The program managers and other stakeholders had substantial understanding on the role of socio-medical determinants. Some of the stakeholders considered healthcare as 'generosity' than individual or collective rights of society, unlike found out in other settings of Africa and Europe [31,32]. The stakeholders from the nutrition and rural development departments expressed their skepticism on the feasibility of monitoring programs involving multiple departments.

"*Giving more focus on tribal and women will make them feel inferior*."[a physician at the state level]

*"Blood is the same for everyone, so why should we have separate monitoring indicators for different social groups?"*[a state level stakeholder]

Key external agencies involved in the policy processes in the Orissa health sector were the World Bank, DFID, UNFPA, UNDP, UNICEF, GFATM and WHO. We found a multi-dimensional relationship among the priorities of those agencies and those of federal and state governments on policy decisions, stemmed from global movements [33]. Some illustrations in this regard are; keeping 'reproductive and child health' as the pivot of NRHM, approaching healthcare through 'disease control programs,' introduction of user fees impacting the poor, reversing the promotion of private sector through 'public-private partnerships'etc. [14]. There is an impression that the grooming of healthcare facilities, human resources, financial incentives, evaluation frameworks, community-based programs and inter-sectoral convergence have been geared for improving maternal and child health status. Other MDG priorities such as combating HIV/AIDS, malaria and tuberculosis also got specific approaches in the healthcare delivery system, indicating the influence of a global momentum locally.

*"It is true that currently, the public health system has been re-designed for improving maternal and child health. I feel, people's other health issues, especially those of women and elderly should be prioritized." *[a state level stakeholder from a development agency]

*"I think almost all developing countries have the same health issues. So, having the same prioritization for healthcare delivery in all these countries is just a coincidence. However, it is a matter of concern that sometimes, global strategies dominate local needs and solutions." *[a state level stakeholder]

Apart from global priorities, the socio-economic, political, religious and cultural factors also had a direct influence on health policies [16]. Addressing inequalities in human development among different social groups and regions is a political mandate in a democratic state like Orissa [14]. For example, the remedial approach was faster for epidemic outbreaks and issues of socio-religious minorities. The large presence of tribal populations also accentuated the scope of policy concerns on health equity as all the policy processes posed specific priorities for such groups. Thus, as we have seen above, the 'actors and context' limited the equity approach in the policy processes and outcomes on account of; information asymmetry among departments and actors involved, non-willingness and less awareness on collective approach on social policies vis-à-vis health of populations, less capacity of actors involved, limited practice of decentralization and involvement of civil society organizations, and multi-dimensional relationship between the mandate of various donor agencies and those of the federal and state governments.

**Figure 2:**
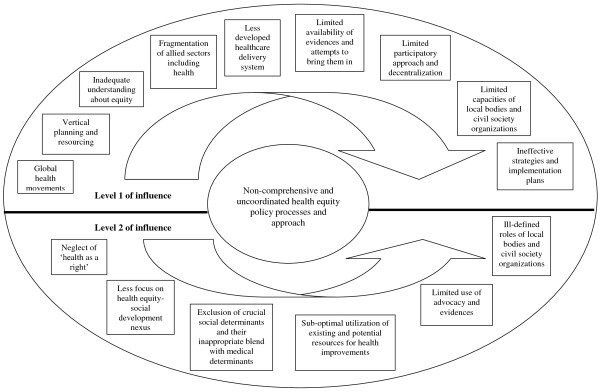
**Nexus of health equity policy decisions and determinants**. Note: The determinants of policy decisions on health equity at level 2 are the direct and indirect outcomes of level 1.

In a nutshell, the policy decision on health equity was an outcome of multiple factors operating in tandem such as; the nature of health equity issues and their perceived importance; existing nature of healthcare delivery system; and global, national, state and district level socio-political, economic and cultural movements. The current influence of the determinants of policy decisions on health equity is described in Figure 2. The figure shows the influence of various determinants for the incomprehensive and uncoordinated health equity policy processes and approach at two levels. The determinants at level 2 are the direct and indirect outcomes of those at level 1.

### Opportunities and policy implications

Orissa has much potential to address health equity, given the scope of ongoing health sector reforms. The current roll-out of OHSP integrated with NRHM seems to be promising as pointed out by the stakeholders. Both technical and financial resources are inevitable to address health equity, which are expected to be enhanced largely through step-wise plans [3]. The role of non-state actors (donors, healthcare institutions, research organizations, media, civil society organizations etc.) is an essential mainstay of any health policy processes.

In retrospective, Orissa achieved considerable healthcare infrastructure improvements with the support of external donor agencies, which is a stepping stone to improve service delivery. The emergence of new hospital buildings, hospital beds and other equipments, and renovation of old buildings are examples in this regard. However, as pointed out by a state level stakeholder, if we look at the cost-effectiveness of those investments being made on infrastructure, the outcome might not be promising. The donors tend to have a multi-faceted approach by focusing on other equity related issues like poverty, livelihoods, water and sanitation, agriculture, irrigation, and development of infrastructure [33]. However, there needs to be an integrated approach for all these efforts among different actors to avoid information asymmetry, duplication of efforts, cater to unmet needs and ensure aid effectiveness. The upcoming federal government initiative on Universal Healthcare Access provision holds scope for Orissa, provided it optimally and specifically customizes for marginalized groups' needs [34].

In the context of Orissa or any other developing health system, the foremost essential requirement of a policy process is to have an explicit equity approach. The proposed checklist to measure the minimum essential requirements of health equity approach in health policy processes is given in Table 2. The checklist can measure the presence of key requirements of policy decisions on health equity at various health policy processes. It also gives some examples of crucial equitable strategies as part of 'policy development' (second stage of policy process) in resource mobilization, allocation, service provision, quality of care, capacity development of stakeholders etc. For instance, it envisages public health regulation, essential service packages, grievance redressal mechanisms etc. as possible necessities.

**Table 2:** Checklist for measuring the minimum key requirements of health equity policy processes:

Agenda setting	Policy development			Implementation and Evaluation
	
	Approach on strategies	Examples of equity centric strategies	Examples of equity centric strategies	
› Specific policy or policy approach on health equity	› Clearly defined participatory, decentralized and bottom-up planning, implementation and evaluation strategies	› Efforts to mobilize and pool people's social, financial and physical resources for social needs (e.g. philanthropies, corporate social responsibility, community resources, social capital etc.)	› Sensitization of all stakeholders on health equity to ensure appropriate policy decisions and accountability	› In-built equity surveillance or indicator based implementation and monitoring of programs
› Acceptance of health as a right	› Strategies for comprehensive inclusion of all vulnerable groups and their needs and differential plans	› Essential service package and quality assurance for vulnerable groups	› Sensitization on health equity in all pre-service and in-service trainings	› Periodical impact evaluation of programs on equity (e.g. Social and Beneficiary Assessment, Benefit-Incidence Analysis etc.)
› Optimal prioritization of diseases, public health interventions and other social determinants	› Defined strategies in mobilizing, pooling and allocation of resources and services based on needs by tapping the potential resources, and using evidences	› Incentives, subsidies or financial risk-protection measures to enhance physical, financial and social access to care	› Capacity development of lower level health facilities to address equity with defined role, resources and monitoring	› Encouragement of exploratory research on health determinants, health outcomes and health policy processes
› Encouragement of participatory approaches	› Defined strategies for inter-sectoral convergence and provision of public health goods	› Public health regulations to ensure quality, affordable and acceptable care (clinical guidelines, price and quality controls, citizens charters) and grievance redressal mechanisms		
› Emphasis on physical, financial and social access to care	› Attempts on integrated planning of strategies, their implementation and evaluation by health and allied departments			
› Focus on evidences for differential planning				
› Integrated approach of health and other social and public policies				

The attempts to review policy processes are challenging in resource constraint settings like Orissa with less documentation of policy approaches and fragmentation of departments. The review did not explore other social and public policies on health equity, but is one of the very few attempts in developing health systems to explore health policy processes. The study applying a scientifically recognized policy analysis matrix, selected respondents purposively as they were at the helm of policy process in the study setting and there were no other sources available. As usual for any such interviews, there was a scope for expressing subjective opinions, but we tried to gather evidences to support opinions of the interviewees and substantiate other arguments. However, the study had access to limited information as the system had been just in the process of recording and documenting various policy processes and outcomes methodically.

## Conclusions

Health equity secured a position in all health processes viz. policy agenda, development, implementation and evaluation to some extent. While, the agenda setting seems to be largely lucrative for equity, the subsequent stages of policy processes faced desertion as the comprehensive issues of vulnerable groups are omitted. The major constraint of a comprehensive approach towards health equity is the nexus among the national and state health priorities, existing weak health care delivery system, and technical and non-technical resource constraints. A common platform to approach on equity is missing owing to; fragmentation of departments, responsibilities and programs; ineffective decentralization; and limited inter-sectoral convergence, evidences on inequalities, participation of civil society organizations, and awareness among stakeholders on equity. The current multi-faceted approach of donors, i.e. equal focus on allied sectors like poverty, nutrition, education, and rural development is encouraging, but all such efforts should be integrated with health sector to avoid information asymmetry and duplication, and improve aid effectiveness.

## Endnotes

^i^Grounded theory approach: validation of stakeholders' opinions or quotes through literature review. Available from: http://findarticles.com/p/articles/mi_m0FSL/is_6_73/ai_75562157/

^ii^Archival analysis: a processing of churning information through older documents or information sources by sorting them in a particular manner with a specific research question. Available from: http://www.psychology-lexicon.com/cms/glossary/glossary-a/archival-analysis.html

## Competing interests

The authors declare that they have no competing interests.

## Authors' contributions

All authors participated in the conceptualization and writing of the manuscript. The primary author wrote the first draft, conducted the interviews and data analysis. The manuscript was read and approved by all authors.

## References

[B1] Braveman P Health disparities and health equity: Concepts and Measurement Annu Rev Public Health 2006 27 167 94 10.1146/annurev.publhealth.27.021405.102103 16533114

[B2] The World Health Organisation Primary Health Care - Now More Than Ever The World Health Report 2008 1 25

[B3] Committee for Development Policy Implementing the Millennium Development Goals: Health Inequality and the Role of Global Health Partnerships United Nations Department of Economic and Social Affairs (DESA) New York 2009 5 12

[B4] Pariyo GW Ekirapa-Kiracho E Okui O Rahman MH Peterson S Bishai DM Lucas H Peters DH Changes in utilization of health services among poor and rural residents in Uganda: are reforms benefitting the poor? Int J Equity Health 2009 8 39 10.1186/1475-9276-8-39 19909514 PMC2781807

[B5] Makwiza I Nyirenda L Bongololo G Banda T Chimzizi R Theobald S Who has access to counseling and testing and anti-retroviral therapy in Malawi - an equity analysis Int J Equity Health 2009 8 13 10.1186/1475-9276-8-13 19416512 PMC2683850

[B6] Grundy J Khut QY Oum S Annear P Ky V Health system strengthening in Cambodia-a case study of health policy response to social transition Health Policy 2009 92 2-3 107 115 10.1016/j.healthpol.2009.05.001 19501425

[B7] Norheim OF Asada Y The ideal of equal health revisited: definitions and measures of inequity in health should be better integrated with theories of distributive justice Int J Equity Health 2009 8 40 10.1186/1475-9276-8-40 19922612 PMC2784761

[B8] Commission on Social Determinants of Health (CSDH) Closing the Gap in a Generation: Health equity through action on the social determinants of health (final report) World Health Organization Geneva 2008 http://whqlibdoc.who.int/publications/2008/9789241563703_eng.pdf

[B9] Chen LC Evans TG Cash RA Kaul I, Grunberg I, Stern MA Health as a global public good Global public goods: international cooperation in the 21st century 1999 Oxford University Press 284 304

[B10] Exworthy M Policy to tackle the social determinants of health: using conceptual models to understand the policy process Health Policy and Planning 2008 23 318 327 10.1093/heapol/czn022 18701553

[B11] C-TRAN Situation analysis of Health Equity in Orissa Report, Government of Orissa 2009 2 23

[B12] Gopalan SS Review of Health Equity in Orissa Report, Government of Orissa 2009 5 29

[B13] Government of India Mission Document National Rural Health Mission. New Delhi 2005 12 35

[B14] Labonte R Schrecker T Towards health-equitable globalization: rights, regulation and redistribution Final Report to the Commission on Social Determinants of Health 2009 Globalization Knowledge Network http://www.who.int/social_determinants/en/

[B15] Lundberg O Yngwe MA Stjärne MK The role of welfare state principles and generosity in social policy programmes for public health: an international comparative study Lancet 2008 372 1633 40 10.1016/S0140-6736(08)61686-4 18994660

[B16] Government of Orissa The Orissa Health Sector Plan (OHSP) Document 2004 2 7

[B17] Walt G Shiffman J Schneider H Murray SF Brugha R Gilson L Doing' health policy analysis: methodological and conceptual reflections and challenges Health Policy and Planning 2008 23 5 308 17 10.1093/heapol/czn024 18701552 PMC2515406

[B18] Kruk ME Freedman LP Assessing health system performance in developing countries: a review of the literature Health Policy 2008 85 3 263 276 10.1016/j.healthpol.2007.09.003 17931736

[B19] Nutbeam D Getting evidence into policy and practice to address health inequalities Health Promotion International 2004 19 137 40 15128705 10.1093/heapro/dah201

[B20] Collins PA Hayes MV The role of urban municipal governments in reducing health inequities: A meta-narrative mapping analysis Int J Equity Health 2010 9 13 10.1186/1475-9276-9-13 20500850 PMC2893183

[B21] Yu CP Whynes DY Sach TH Reform towards National Health Insurance in Malaysia: The equity implications Health Policy 2011 100 256 263 10.1016/j.healthpol.2010.10.018 21129808

[B22] Salti N Chaaban J Raad F Health equity in Lebanon: a microeconomic analysis Int J Equity Health 2010 9 1 10.1186/1475-9276-9-1 20398278 PMC2864280

[B23] Williams JA Byles JE Inder KJ Equity of access to cardiac rehabilitation: the role of system factors Int J Equity Health 2010 9 2 10.1186/1475-9276-9-2 20205776 PMC2823593

[B24] What are the effects of health care reforms on gender equity, particularly in health? J Health Serv Res Policy 2006 11 4 255 17018203 10.1258/135581906778476599

[B25] Soderlund N Possible objectives and resulting entitlements of essential health care packages Health Policy 1998 45 3 195 208 10.1016/S0168-8510(98)00039-6 10338951

[B26] Musgrove P Public spending on health care: how are different criteria related? Health Policy 1999 47 3 207 223 10.1016/S0168-8510(99)00024-X 10538919

[B27] Oyaya CO Rifkin SB Health sector reforms in Kenya: an examination of district level planning Health Policy 2003 64 1 113 127 10.1016/S0168-8510(02)00164-1 12644333

[B28] Hyder AA Corluka A Winch PJ National policy-makers speak out: are researchers giving them what they need? Health Policy and Planning 2011 26 73 82 10.1093/heapol/czq020 20547652 PMC4031573

[B29] Freeman R The work the document does: research, policy and equity in health Journal of Health Policy, Politics and Law 2006 31 51 70 10.1215/03616878-31-1-51 16484668

[B30] Sundewall J Forsberg BC Tomson G Theory and practice - a case study of coordination and ownership in the Bangladesh health SWAp Health Research Policy and Systems 2006 4 5 10.1186/1478-4505-4-5 16704726 PMC1479819

[B31] Ratcliffe J Bekker HL Dolan P Edlin R Examining the attitudes and preferences of health care decision-makers in relation to access, equity and cost-effectiveness: a discrete choice experiment Health Policy 2009 90 1 45 57 10.1016/j.healthpol.2008.09.001 18937994

[B32] Ottersen T Mbilinyi D Maestad O Norheim OF Distribution matters: equity considerations among health planners in Tanzania Health Policy 2008 85 2 218 227 10.1016/j.healthpol.2007.07.012 17825939

[B33] Sridhar D Gomez EJ Health Financing in Brazil, Russia and India: What Role Does the International Community Play? Health Policy and Planning 2011 26 12 24 10.1093/heapol/czq016 20400535

[B34] Balarajan Y Selvaraj S Subramanian SV Healthcare and equity in India The Lancet 2011 377 9764 505 515 10.1016/S0140-6736(10)61894-6 PMC3093249 21227492

